# Supplementary material for: In Vitro Characterization of the Human Skeletal Stem Cell-like Properties of Primary Bone-Derived Mesenchymal Stem/Stromal Cells in Patients with Late and Early Hip Osteoarthritis
Source: Life (Basel). 2022 Jun 15;12(6):899. doi: 10.3390/life12060899 (PMC9228448; doi:10.3390/life12060899)
Supplement: Supplementary file 1 [file life-12-00899-s001.zip › life-1738936-supplementary.pdf]

Supplementary Figures.

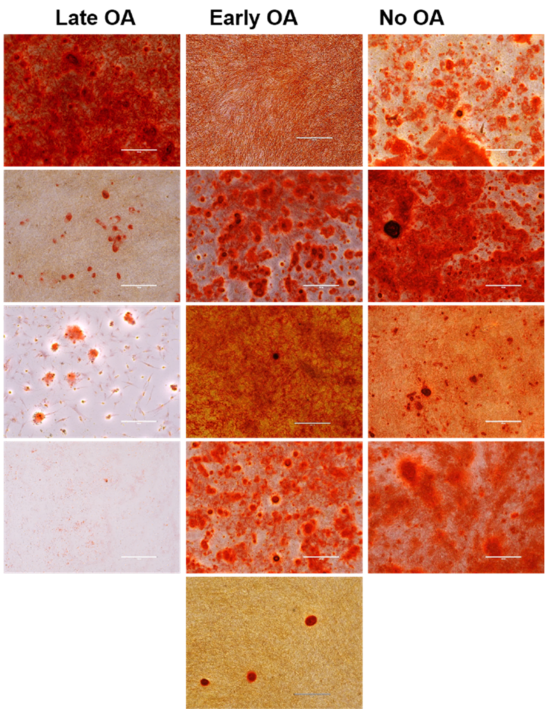

Supplemental Figure S1. The images of the wells for each donor in all groups (as indicated) stained with Alizarin red S for osteogenesis. Scale bars, 400 μm. OA, osteoarthritis.

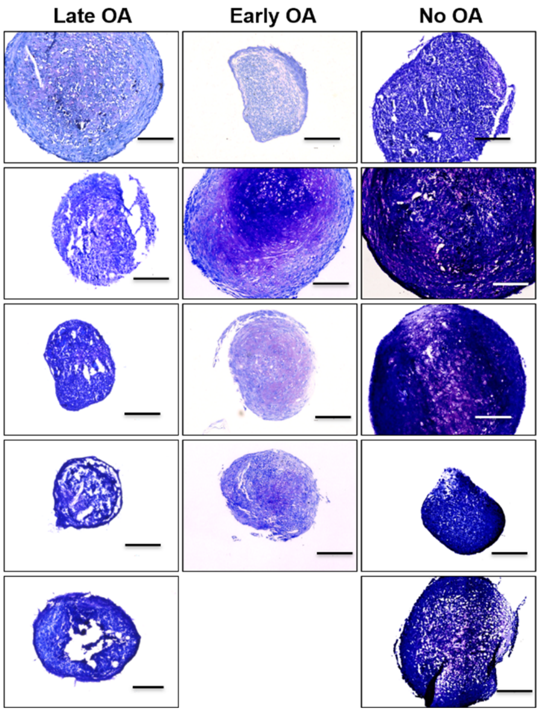

Supplemental Figure S2. The images of the wells for each donor in all groups (as indicated) stained with Toluidine blue for chondrogenesis. Scale bars, 200 μm. OA, osteoarthritis.

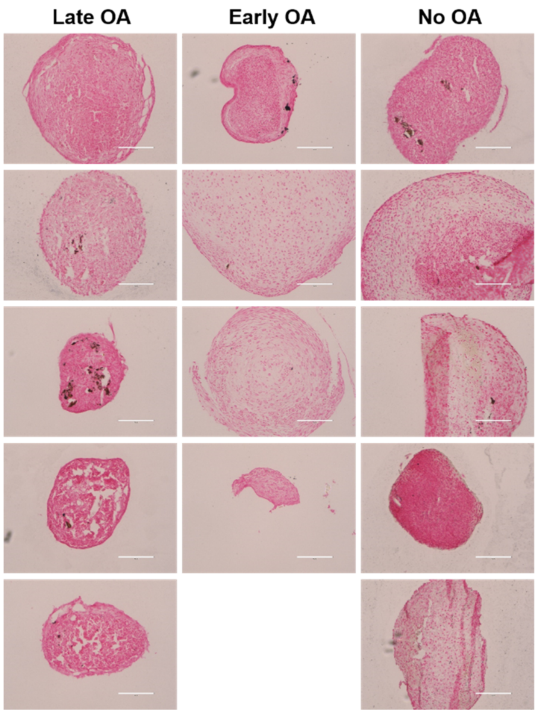

Supplemental Figure S3. The images of the wells for each donor in all groups (as indicated) stained with von Kossa for the mineralization rate in chondrogenesis.
Scale bars, 200 μm. OA, osteoarthritis.

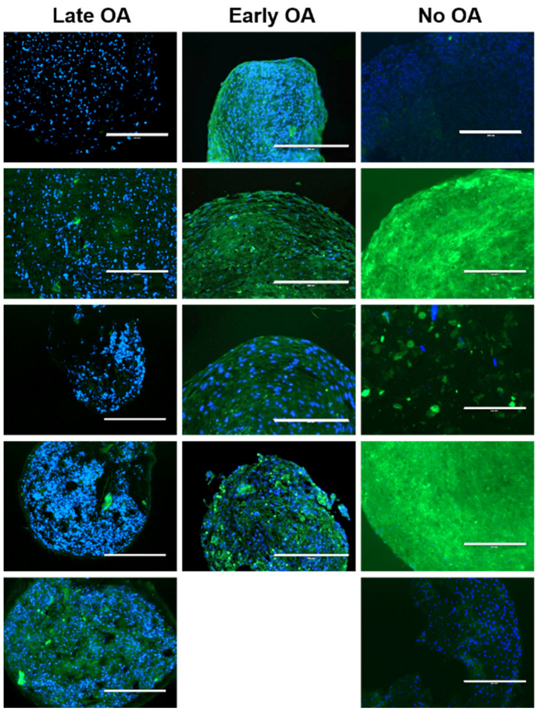

Supplemental Figure S4. The images of the wells for each donor in all groups (as indicated) showing immunofluorescence for the α-1 chain of type II collagen (Col2A1).
Scale bars, 200 μm. OA, osteoarthritis.

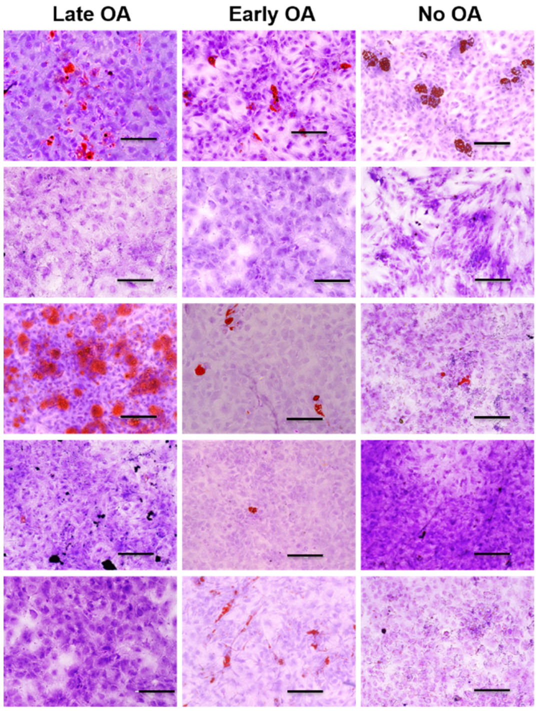

Supplemental Figure S5. The images of the wells for each donor in all groups (as indicated) showing Oil red O stained adipocytes. Scale bars, 200 μm. OA, osteoarthritis.
